# Pathogenesis of Focal Segmental Glomerulosclerosis and Minimal Change Disease: Insights from Glomerular Proteomics

**DOI:** 10.3390/life15040527

**Published:** 2025-03-23

**Authors:** Yuriy Maslyennikov, Ioana-Ecaterina Pralea, Andrada Alina Bărar, Crina Claudia Rusu, Diana Tania Moldovan, Alina Ramona Potra, Dacian Tirinescu, Maria Țicală, Alexandra Urs, Paula Zamfir, Emil Boțan, Ximena-Maria Mureșan, Simina Pîrv, Andreea Nuțu, Ioana Berindan-Neagoe, Cristina-Adela Iuga, Ina Maria Kacso

**Affiliations:** 1 Department of Nephrology, Faculty of Medicine, “Iuliu Hațieganu” University of Medicine and Pharmacy, 400349 Cluj-Napoca, Romania; maslyennikov_yuriy@elearn.umfcluj.ro (Y.M.); barar_andrada_alina@elearn.umfcluj.ro (A.A.B.); claudia.rusu@umfcluj.ro (C.C.R.); diana.moldovan@umfcluj.ro (D.T.M.); alina.potra@umfcluj.ro (A.R.P.); tirinescu.dacian@umfcluj.ro (D.T.); cosa.maria@umfcluj.ro (M.Ț.); alexandra.urs@elearn.umfcluj.ro (A.U.); maria.kacso@umfcluj.ro (I.M.K.); 2 Personalized Medicine and Rare Diseases Department, MEDFUTURE—Institute for Biomedical Research, “Iuliu Hațieganu” University of Medicine and Pharmacy, 400349 Cluj-Napoca, Romaniaximena.muresan@medfuture.ro (X.-M.M.); simina.pirv@medfuture.ro (S.P.); 3 Department of Pathology, Regional Institute of Gastroenterology and Hepatology, 400162 Cluj-Napoca, Romania; paulacristinela@yahoo.com; 4 Department of Pathology, County Emergency Hospital Cluj-Napoca, 400347 Cluj-Napoca, Romania; emilbotan@gmail.com; 5 Genomics Department, Institute for Biomedical Research, “Iuliu Hațieganu” University of Medicine and Pharmacy, 400349 Cluj-Napoca, Romania; andreeanutu.an@gmail.com (A.N.); ioananeagoe29@gmail.com (I.B.-N.); 6 Department of Pharmaceutical Analysis, Faculty of Pharmacy, “Iuliu Hațieganu” University of Medicine and Pharmacy, 400349 Cluj-Napoca, Romania

**Keywords:** focal segmental glomerulosclerosis, minimal change disease, proteomics, tissue, glomeruli

## Abstract

Podocyte injury is a hallmark of both focal segmental glomerulosclerosis (FSGS) and minimal change disease (MCD), ultimately reflected in foot process effacement and proteinuria. Triggers and pathogenic pathways leading to podocyte cytoskeleton rearrangements are, however, incompletely explained. Here, we aimed to contribute to the understanding of these pathways using tissue bottom-up proteomic profiling of laser-capture microdissected glomeruli from MCD and FSGS. Forty-six differentially expressed proteins were identified between the two groups (*p* < 0.05, |FC| ≥ 1.2). Pathway analysis showed that 16 out of 46 proteins were associated with the immune system, with E2 ubiquitin-conjugating enzyme (UBE2K) and complement factor H-related protein-1 (CFHR1) yielding the highest fold change in FSGS compared to MCD. The two target proteins were further validated through immunohistochemistry, confirming the podocyte localization of UBE2K and endothelial staining of CFHR. Additionally, several other differentially expressed proteins were linked to the cytoskeleton structure and its regulation. Our results point to the possibility that complement dysregulation may be the source of cytoskeleton rearrangement in FSGS.

## 1. Introduction

Podocytes, visceral epithelial cells that line the outer aspect of the glomerular basement membrane (GBM), contribute decisively to the permselectivity of the glomerular filtration. They have a complex branching aspect which allows for the development of specialized structures essential for selective filtration of plasma—the slit diaphragms (SDs). These are formed between third-degree foot processes (FPs) of neighboring podocytes and consist of specialized proteins, such as nephrin, podocin, and others. Podocytes are anchored to the GBM by special structures known as focal adhesion (FA). Both FA and SD are important anchoring points but also main signaling hubs that define podocyte stability and function [1,2]. The podocytes have a complex cytoskeleton: mainly microtubules and intermediate filaments are seen in the cell body and primary cell processes, whereas actin, myosin, and alpha-actinin predominate in tertiary FP [3]. Rearrangement of the podocyte cytoskeleton occurs in response to injury, mediated by various triggers; these rearrangements result in foot process effacement (FPE) and proteinuria. As podocytes are terminally differentiated cells and do not divide, extensive injury may result in detachment from the GBM with denudation of the latter [4].

Podocytopathies are glomerular diseases manifested as nephrotic syndrome that arise from dysfunction and/or injury of podocytes and are characterized by FPE seen on electron microscopy. The main histological forms are minimal change disease (MCD) and focal segmental glomerulosclerosis (FSGS), which present in most instances with a similar clinical picture. However, the underlying mechanism might be very different, as is the case with a response to therapy. In MCD, the only histologic change is the diffuse effacement of FP, while a common characteristic is the positive response to corticotherapy. Nevertheless, the mechanisms involved are not uniform and might be related to a variety of factors (genetic causes, toxic injury, lymphocyte B and T dysfunction) [5]. In FSGS, the histological pattern is characterized by matrix accumulation and sclerosis, podocyte–epithelial apposition, and, sometimes, proliferation along with various degrees of FPE [6]. This pattern is also associated with different etiologies and pathogenic pathways, such as intraglomerular hyperpressure and hyperfiltration, viral infection, drug toxicity, genetic factors, or unknown causes [7]. Response to corticotherapy in FSGS is less uniform, outcomes are largely unpredictable, and attempts at histological classification of FSGS [8,9] did not result in improved prognostic information. Among all glomerular diseases that manifest with nephrotic syndrome, FSGS probably holds the worst prognosis. To provide urgently needed and personalized treatment for patients with podocytopathies, it is mandatory to understand the mechanisms of podocyte injury and the pathogenic pathways involved both in MCD and FSGS.

Over the past decade, laser microdissection of glomeruli, coupled with advances in proteomic techniques, provided valuable insights into glomerular diseases that have been, in some cases, groundbreaking. For example, proteomic findings have played a pivotal role in reclassifying membranoproliferative glomerulonephritis (MPGN) according to pathogenesis [10], enabling the development of a more targeted treatment approach. Proteomic studies have also led to major advances in untangling the mechanisms of injury in membranous nephropathy [11]. These insights have fundamentally changed the diagnosis process and therapeutic approaches, starting with the description of anti-phospholipase A2 receptor antibody [12] over a whole more disease-specific spectrum of antigens. Major advances have also expanded knowledge in amyloidosis with a description of new antigens [13]. Proteomic glomerular studies generally require few patients to yield significant results [14,15]. Despite this, attempts to describe MCD and FSGS using tissue proteomic studies have been limited [16,17,18,19], highlighting the need for more research to better understand these diseases and their underlying mechanisms.

To deepen our understanding of the mechanisms driving the pathophysiology of FSGS, our study focused on comparing mass spectrometry proteomic data obtained from laser-dissected glomeruli extracted from formalin-fixed paraffin-embedded (FFPE) tissues of patients diagnosed with either FSGS or MCD. This approach aimed to uncover specific protein profiles that could reveal the distinct molecular features of these two kidney disorders.

## 2. Materials and Methods

### 2.1. Sample Collection and Storage

We conducted a retrospective, observational, and cross-sectional study on FSGS and MCD. Formalin-fixed paraffin-embedded renal tissue was collected from County Emergency Hospital Cluj-Napoca between 2019 and 2022. Biopsy tissue samples were analyzed by the hospital pathologist, who confirmed the diagnosis of FSGS and MCD using light microscopy, immunofluorescence, and electron microscopy. After obtaining written consent, patients without immunosuppressive medication in the last 12 months and enough leftover FFPE tissue following diagnosis were included in the study.

This study complies with the Declaration of Helsinki and was conducted in accordance with the ethics committee guidelines of Emergency County Hospital Cluj-Napoca (3/2021) and “Iuliu Hațieganu” University of Medicine and Pharmacy Cluj-Napoca (142/2021).

For FSGS patients, TEM was performed in order to quantify the degree of FPE. Focal FPE (defined as less than 80% of glomerular circumference affected), together with clinical data (such as obesity and drug toxicity), allowed the identification of secondary causes of FSGS, which were excluded from this study. However, the genetic analysis was not performed in this cohort. All tissue samples subjected to proteomic analysis were taken at the presentation. Data regarding previously diagnosed nephrotic syndrome and immunosuppressive medication were recorded. Clinical and paraclinical data were collected for each patient from the electronic records of County Emergency Hospital Cluj-Napoca and consisted of sex, age, serum creatinine, estimated glomerular filtration rate (eGFR), serum albumin, total serum proteins, total cholesterol, hemoglobin, presence of hematuria (defined as repeatedly >10 erythrocytes/high power field), and urinary 24 h protein excretion. The prescribed immunosuppression regimen(s) and response to therapy, as well as the length of follow-up, were retrieved from the records. Remission was defined according to the latest KDIGO guidelines [7] as complete remission in patients with a reduction in proteinuria to <0.3 g/24 h, stable renal function and serum albumin > 3.5 g/dL, and partial remission in patients with a reduction in proteinuria to 0.3–3.5 g/24 h and a decrease > 50% from baseline. Relapse was defined as a reappearance of nephrotic syndrome after remission.

IBM SPSS version 25.0 was utilized to conduct statistical analysis of clinical and paraclinical data. Normally distributed quantitative variables were expressed as arithmetic mean ± standard deviation or percentages, where applicable. In contrast, not normally distributed variables were reported as median along with the interquartile range (the 25th–75th percentiles). Rank comparisons were performed using the *t*-test for normally distributed data and the Mann–Whitney–Wilcoxon test for data following abnormal distribution. Variance comparisons were conducted using Levene’s test. Dichotomous variables were presented as absolute frequency along with their relative percentage frequency. These types of variables were analyzed using the Fisher exact test. The significance threshold for all tests conducted was set at α = 0.05.

### 2.2. Samples Preparation for Bottom-Up Proteomics Profiling

The methodology established by Bărar et al. [16] was adapted for the current study. Briefly, 10 µm tissue sections were mounted on MMI RNase/DNase-free membrane slides (Molecular Machines & Industries, Eching, Germany) and subjected to staining according to Mayer’s hematoxylin protocol. Glomerular sections were then isolated using the MMI CellCut Plus laser capture microdissection system (MMI AG, Glattbrugg, Switzerland). The isolated laser capture microdissection (LCM) samples were resuspended in 0.1% Rapigest (prepared in 100 mM ammonium bicarbonate, Waters Corporation, Milford, MA, USA) and placed into protein low-retention tubes. Protein extraction was performed via sonication (3 × 3 s, 80% amplitude) and subsequent heating steps (95 °C for 15 min and 80 °C for 60 min). For further sample processing, a single-pot solid-phase-enhanced sample preparation (SP3) protocol was employed, utilizing two types of carboxylate-modified paramagnetic beads (Sera-Mag SpeedBeads, GE Healthcare, Chicago, IL, USA), as described by Hughes et al. [20]. Peptides were eluted from the beads using 2% DMSO (*v*/*v* in water), and the resulting supernatant was transferred to a new vial and acidified with 1% formic acid. For each biological sample, two technical replicates of the same protein amount were processed using the SP3-aided sample preparation procedure followed by LC-MS analysis. The technical replicates were performed under identical conditions to assess the variability and reproducibility of the entire protocol.

### 2.3. LC-MS Analysis

LC-MS analyses, data processing, and label-free quantification were carried out in accordance with the methodologies described in a prior publication [16]. In summary, LC−MS analyses were performed using a NanoAQUITY UPLC system (Waters Corporation, Milford, MA, USA) coupled with a Synapt G2-S mass spectrometer (Waters Corporation, Wilmslow, UK) via NanoLockSpray dual electrospray ion source. Water containing 0.1% (*v*/*v*) formic acid was used as mobile phase A, while acetonitrile containing 0.1% (*v*/*v*) formic acid was used as mobile phase B. After injecting 2 µL of sample, peptides were trapped for 2 min on an ACQUITY UPLC M-Class Trap Symmetry C18 column (5 μm, 180 μm × 20 mm) and separated on a reverse-phase C18 HSS T3 column (1.8 μm, 75 μm × 250 mm) using a 45 min gradient from 5% to 85% solvent B at a flow rate of 300 nL/minute. Following separation, the column was rinsed for 3 min with 85% solvent B and then re-equilibrated to initial conditions for 17 min. The column temperature was set at 55 °C throughout the analysis.

MS acquisition was performed in UDMSE mode with ion-mobility separation (IMS)-enhanced data-independent acquisition (DIA) as previously described by Distler et al. [21]. Briefly, all MS measurements were recorded over a mass range of 50 to 2000 *m*/*z*, using a scan time of 0.6 s, with the instrument configured in resolution mode and employing positive ion mode electrospray with a typical resolving power of at least 22,000 FWHM (full width at half maximum). Lock mass (Glu-1-fibrinopeptide B) was delivered at 0.3 μL/min, and spectra were recorded every 45 s. The following source settings were used: 90 °C source temperature, 30 V sampling cone voltage, 3.0 kV capillary voltage, and 80 V source offset. The Step Wave settings in TOF mobility acquisition mode were as follows: the wave velocity was set at 300 m/s, while the wave heights were set to 10 V, 5 V, and 0 V for the StepWave 1, StepWave 2, and Source Ion Guide, respectively. The trapping settings for the ion mobility mode were set as follows: 500 µs for Mobility Trapping Release Time, 15.0 V for Mobility Trap Height, 0 V for Mobility Extract Height, and 450 µs for IMS wave delay. For IMS, the wave height was set to 40 V with a velocity of 650 m/s. In the trap and transfer cell, wave velocities were set to 313 m/s and 190 m/s, while the wave heights were set to 8 V and 4 V, respectively. Additionally, the IMS wave velocity was ramped during the full IMS cycle, with a starting velocity of 800 m/s and an ending velocity of 500 m/s. In the UDMSE acquisition, no collision energy (CE) was applied to the trap and transfer for the low energy scan. For the high energy scans, a look-up table file was utilized to ramp the collision energy in the transfer cell. The following collision energy settings were applied throughout the study for the elevated energy scans: (i) Ion-mobility bins 0–20: CE of 4 eV; (ii) Ion-mobility bins 21–190: CE ramped from 17 eV to 75 eV; (iii) Ion-mobility bins 191–200: CE of 4 eV.

Raw mass spectrometry data were analyzed using Progenesis QIP 4.2 (Waters Corporation, Milford, MA, USA), which involved applying lock–mass correction with the doubly charged monoisotopic ion of GluFib and aligning the data to the most appropriate reference run performed automatically by the software. The normalization of all protein options was utilized. Identified proteins were matched against the UniProtKB/Swiss-Prot Human target-decoy database (20,361 proteins, January 2022) using the following search parameters: (i) trypsin digestion with a maximum of one missed cleavage; (ii) fixed modifications: carbamidomethylating and methionine oxidation; (iii) variable modifications: hydroxylation of asparagine, methylation of aspartic acid, proline, lysine, as well as lysine methylation and formylation at the N-terminus. The false discovery rate (FDR) was established at less than 1%. The criteria for ion matching required at least one fragment ion per peptide, three fragment ions per protein, and a minimum of one peptide match per protein (1-3-1). Peptides exhibiting mass errors greater than 20 ppm or containing fewer than five amino acids were excluded from the analysis.

### 2.4. Differential Protein Abundance Analysis

The dataset obtained from Progenesis QIP for proteomics was filtered to exclude reverse sequences, and technical replicates were averaged. Post-processing protein analysis was performed using the MetaboAnalyst 6.0 Statistical Analysis module (one-factor analysis) [22] (accessed on 17 June 2024). For differential abundance analysis, a two-sample *t*-test was applied. Proteins were considered significantly altered if both the *p*-value was ≤0.05 and the fold change (|FC|) was ≥1.2. Data visualizations, including PCA plots and heatmaps, were generated utilizing the same online tool. Standard gene nomenclature was employed for the annotation of proteins.

### 2.5. Network Visualization and Enrichment Analysis

Protein–protein interaction (PPI) and enrichment analyses were conducted using STRING (version 12; available at https://string-db.org/, accessed on 17 February 2025), a widely used online tool for functional association and pathway enrichment analysis. The STRING network was retrieved for the differentially expressed proteins using the following parameters: (i) a full STRING network of functional associations and the physical subnetwork was retrieved; (ii) a minimum required interaction score of 0.4; (iii) and only for the query proteins, no added interactors. Functional enrichment analysis was performed using the same online tool, which utilized the Gene Ontology and Reactome pathway databases. A term was considered enriched if the false discovery rate (FDR) associated with the enrichment analysis was less than 0.05, and a minimum of three proteins were identified as being associated with the term.

### 2.6. Immunohistochemistry

Prior to performing sections for laser capture microdissection, 3 µm thick sections were cut from paraffin-embedded blocks and mounted on saline-coated slides. Immunohistochemistry was performed using a Leica Bond Max autostainer (Leica BOND, Leica Byosistems, Deer Park, IL, USA) with an anti-CFHR1 antibody (HPA038922, Sigma Aldrich, St. Louis, MO, USA) and anti-UBE2K antibody (HPA028869, Sigma Aldrich) according to the manufacturer’s instructions. An immunohistochemistry study was performed for the two proteins with the highest positive fold change when analyzed using two different *p*-value thresholds (*p* = 0.05 and *p* = 0.01). The obtained images were processed using Fiji software v2.16.0 [23].

## 3. Results

### 3.1. Comparative Proteomic Analysis of Glomeruli in FSGS and MCD Patients

A total of 12 patients were included in this study, with 6 assigned to the MCD group. The baseline characteristics are detailed in Table 1. Notable differences between groups were only seen in the estimated glomerular filtration rate. No statistically significant differences were observed for the other clinical parameters. In the MCD group, 2 out of 6 patients, MP1 and MP4, had a relapsing course of nephrotic syndrome from childhood and previously underwent treatment with immunosuppressive therapy; however, they did not receive immunosuppressors within 12 months before kidney biopsy. The rest of the patients from the MCD and FSGS groups were newly diagnosed (Table 1). In MCD, first-line immunosuppressive treatment was corticotherapy for all patients; patients MP2-6 responded to this treatment; patient MP1 responded to calcineurin inhibitors (CNIs). Patients MP2 and MP5 had a relapsing course after corticotherapy and responded to the second or third line of therapy with CNI or cyclophosphamide (CFS). All patients maintained stable renal function. In FSGS, the first line of treatment was corticotherapy. Patients GP9 and GP13 responded without relapse; in patient GP7, the response was achieved, but proteinuria relapsed after corticoid withdrawal. Patients GP7 and GP8 responded to the second-line treatment with CNI.

**Figure 1:**
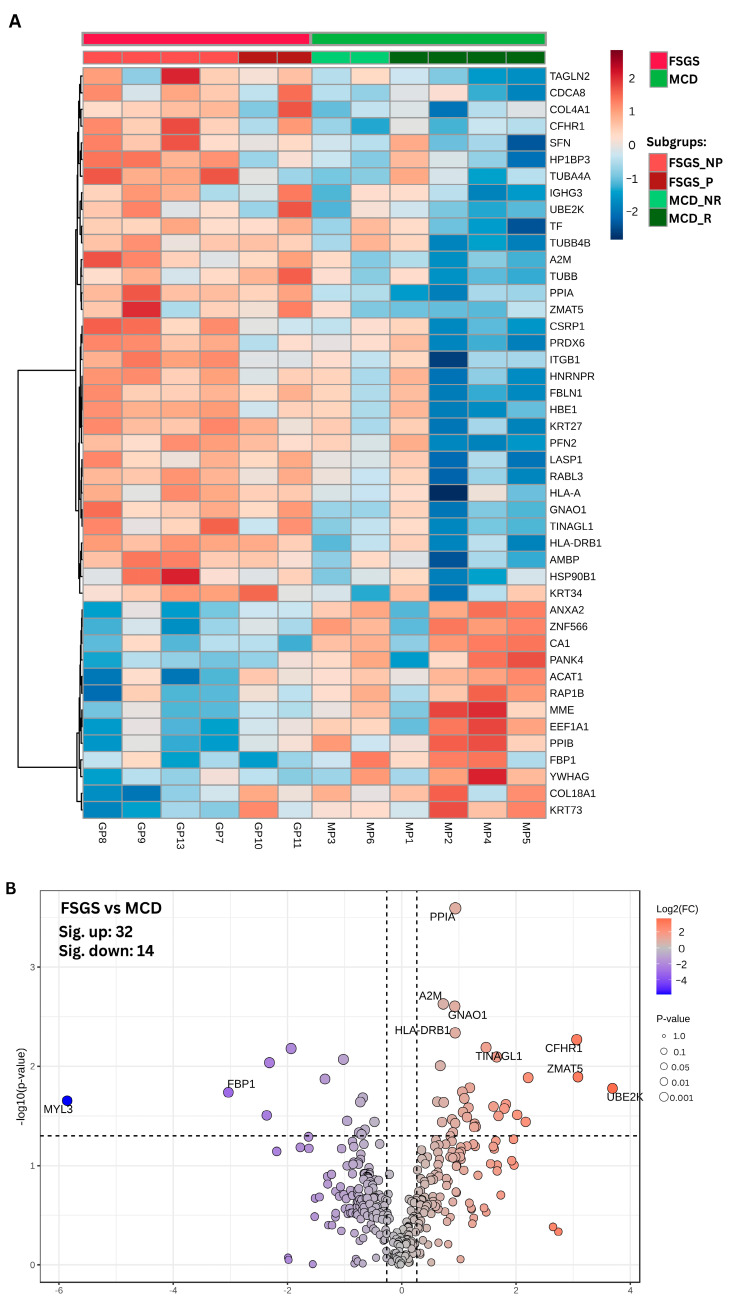
Differential expression analysis results in FSGS vs. MCD groups. (**A**) Heatmap of differentially expressed proteins (unpaired *t*-test, unequal variance, *p* ≤ 0.05; Euclidean distance measure). (**B**) Volcano plot illustrating the differentially expressed proteins (*t*-test, independent unequal variance, *p* ≤ 0.05 and |FC| > 1.2) in the FSGS group compared to the MCD group. Red—DEPs with higher abundance in FSGS; Blue—DEPs with higher abundance in MCD; gray—proteins with no significantly different abundance. Gene names are provided for the top 10 DEPs.

Complete remission was noted in patients MP2-4 and GP9. Partial remission was achieved in the rest of the patients, except for GP10 and GP11, who were classified as having resistant disease without remission and with progression to end-stage renal disease (ESRD) (Table 1). Due to the limited number of patients, subgroup statistical analysis could not be performed; a heatmap of proteomic profiles shows patients grouped according to their response to treatment (Figure 1).

### 3.2. Differently Expressed Proteins

A total of 357 proteins were identified, revealing 46 notable features when comparing the FSGS and MCD groups based on a *p*-value threshold of 0.05 (Figure 1A,B). Among these, 32 proteins demonstrated elevated expression levels in the FSGS group, whereas 14 proteins exhibited decreased expression relative to patients with MCD (Supplementary Table S1). Eleven proteins were identified as differentially expressed at a more stringent *p*-value threshold of 0.01, with all showing fold change values (|FC|) greater than 1.2 (Supplementary Table S1, Supplementary Figure S1). In this comparison between FSGS and MCD groups, the ubiquitin-conjugating enzyme E2 K (UBE2K) exhibited the highest positive fold change of 12.88, meeting the *p*-value threshold of 0.05. Upon adjusting the *p*-value threshold to 0.01, complement factor H-related 1 (CFHR1) emerged as having the highest positive fold change at 8.35. The Human Proteome Atlas database, version 24.0 (proteinatlas.org; accessed on 30 October 2024), was utilized to gather information on proteins that exhibit differential expression [22]. According to the HPA (Supplementary Table S2), 35 of these proteins are present in the blood, indicating their potential as biomarkers, including UBE2K and CFHR1. Additionally, 24 of these proteins have documented expression data in human kidney tissue, specifically in all annotated cell types, with a focus on glomerular cells. Notably, three proteins—ACAT1, MME, and FBP1—with the highest expression levels observed in minimal change disease (MCD) showed kidney tissue specificity.

**Figure 2:**
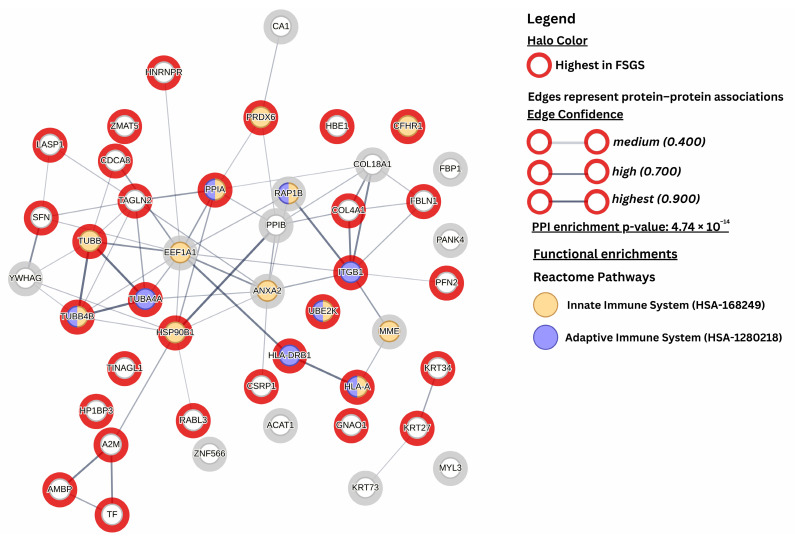
STRING protein–protein interaction network and functional enrichment analysis. This figure illustrates the STRING protein–protein interaction network for the differentially expressed proteins (DEPs) identified between FSGS and MCD, with each node representing a DEP. The network was generated using a minimum required interaction score of 0.4, including only the query proteins (DEPs) and excluding any additional interactors. The confidence of protein–protein associations is depicted by the edge color, ranging from light to dark gray. The halo color around each node indicates the regulation trend: red denotes proteins with higher expression in FSGS, while gray represents proteins with lower expression in FSGS. Node colors reflect the functional enrichment analysis results based on the Reactome database, with DEPs associated with specific pathways. They are color-coded yellow for the innate immune system and blue for the adaptive immune system.

The protein–protein interaction network generated within STRING (accessed on 22 January 2025) revealed a higher level of interaction among the DEPs than would be expected for a random set of proteins of the same size and degree of distribution). This was reflected by a PPI enrichment score of 4.74 × 10^−14^ for the set of 46 DEPs (Figure 2). String functional enrichment analysis (FDR ≤ 0.05, minimum three proteins/pathway) revealed several significant pathways and associated functions, including cytoskeleton organization (GO:0007010, 13 proteins, FDR= 3.91 × 10^−2^) (Supplementary Figure S2), focal adhesion (GO:0005925, 8 proteins, FDR = 7.80 × 10^−4^) (Supplementary Figure S3), cell adhesion molecule binding (GO:0050839, 8 proteins, FDR = 3.58 × 10^−2^) (Supplementary Figure S4), Innate Immune System (HSA-168249, 12 proteins, FDR = 2.20 × 10^−3^), and RHO GTPase Effectors (HSA-195258, 7 proteins, FDR = 2.20 × 10^−3^). A complete list is provided in the Supplementary Table S3.

### 3.3. Immunohistochemistry Results

**Figure 3:**
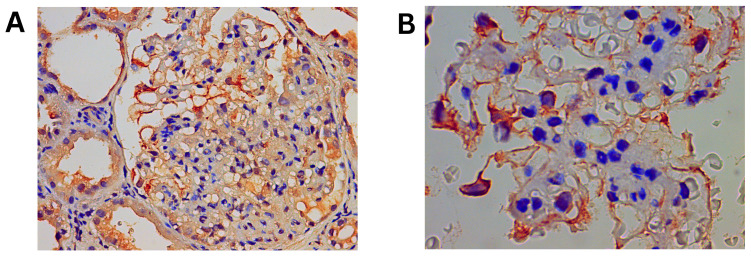
Immunohistochemical UBE2K staining of the kidney biopsies. (**A**) At 40× magnification: moderate/intense glomerular staining. (**B**) At 100× magnification: podocyte cytoplasmatic staining.

**Figure 4:**
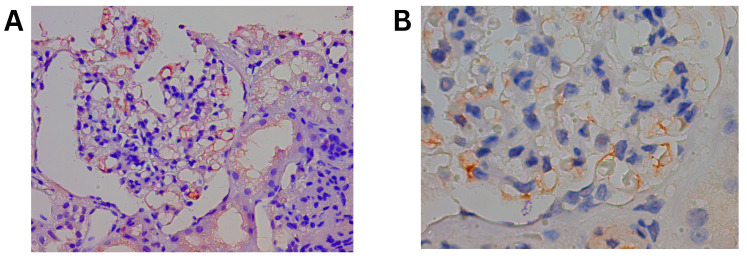
Immunohistochemical CFHR1 staining of the kidney biopsies. (**A**) At 40× magnification: low/moderate intensity glomerular staining. (**B**) At 100× magnification: endothelial staining.

Validation of differentially expressed proteins was performed using immunohistochemistry for the two proteins with the highest positive fold change, UBE2K and CFHR1, in 5 of the 6 FSGS cases. Representative images are shown in Figure 3 and Figure 4. UBE2K staining was positive in 5 of the 5 studied cases at moderate/high intensity, and deposition was primarily confined to the podocyte cell body (Figure 3). CFHR1 was positive in 3 of the 5 cases at weak/moderate intensity with endocapillary deposition. (Figure 4).

## 4. Discussion

Our study is one of the few that addresses the proteomic profile of FSGS for human glomerular tissue. Although proteomic data from human glomerular tissue have significant potential to enhance our understanding of the pathogenic mechanisms underlying FSGS, very few reports have addressed this topic and matrix. One such study investigated the extracellular matrix (ECM) protein fraction in glomeruli from biopsy specimens of patients with FSGS not otherwise specified (FSGS-NOS) and collapsing FSGS [18], while another study analyzed the proteomic profile in leftover kidney biopsy tissue (not micro-dissected glomeruli) in pediatric FSGS [19].

One of our most interesting findings involves complement regulation. Complement activation plays a crucial role in the pathogenesis of several glomerular diseases. However, there are few kidney diseases in which decreased serum complement components (C3 or C4) can be used as diagnostic biomarkers or as indicators of disease progression. This is the case in lupus nephritis, atypical hemolytic uremic syndrome, and MPGN (infection-related C3 glomerulonephritis). In many other instances, complement activation is known to play a pathogenic role (such as in membranous nephropathy and IgA nephropathy) but is only recognized in the immunofluorescence staining of kidney biopsy, and serum levels are normal. There are conditions in which kidney complement deposition is not prominent (such as in ANCA vasculitis), yet the role of complement in pathogenesis and its blockade in management is recognized [25,26]. This might also be the case of FSGS, in which complement staining, traditionally perceived as non-specific, might instead be the consequence of an insufficiently recognized complement-mediated injury. There is mounting evidence for this hypothesis: podocytes have been proven to synthesize complement factors and inhibitors, and they [27] may initiate C3 cleavage and, hence, alternative pathway activation [28,29]. Conversely, podocytes can also be a target of complement activation that induces cytoskeleton rearrangements and glomerulosclerosis [30]. In clinical settings, complement consumption and increased membrane attack complex (MAC) in serum and urine were reported in FSGS patients, correlating with outcomes in terms of proteinuria and renal function [31,32,33,34]. In immunohistochemistry, complement activation was reported in FSGS via lectin pathway, including staining of non-sclerotic glomeruli [35]. Evidence of classical [36] and alternative [30] pathways activation have also been suggested.

GBM protection against complement overactivation is completely dependent on factor H because it is the only terminal complement pathway inhibitor found in the GBM anchored to glycans within the membrane [13,31,37]. Factor H enhances factor I-mediated proteolysis and acts as a decay-accelerating factor for C3Bb [38]. Complement factor H-related protein-1 (CFHR2-5 proteins) have a similar C terminal structure as factor H but lack the N terminal inhibitory amino acid unit; they compete with factor H for complement binding sites on C3b [39,40], disrupting normal complement regulation. Recent data demonstrate that increased CFHR1 is found in active IgA nephropathy, ANCA vasculitis, C3 glomerulonephritis, and atypical hemolytic uremic syndrome and might be associated with CFHR1 gene variants [41,42]. Our findings are important for contributing to the insufficiently characterized landscape of complement activity in FSGS. So far, the only other proteomic study conducted on kidney tissue from pediatric biopsies indicates an increase in terminal complement proteins in FSGS compared to MCD. However, not only the glomeruli but also the whole leftover tissue was analyzed in this study, and validation was not performed for the identified proteins [19]. Due to insufficient data, our finding that CFHR1 is increased in FSGS may be a relevant argument for the importance of alternative pathway dysregulation. In contrast, while complement activation is conceivable in MCD [43], data regarding complement activation derived from serum measurements in MCD are inconsistent [25,44].

Alongside anaphylatoxins C3a and C5a, the main effector of the activated terminal complement pathway is the MAC. While the first and well-recognized role of the MAC, if present in sufficient amounts, is to mediate cell lysis, there is increasing evidence that sub-lytic levels of MAC can trigger other intracellular processes in exposed cells that influence cytoskeleton architecture, cell proliferation, motility, the secretion of pro-inflammatory cytokine synthesis and, ultimately, apoptosis [45,46]. One of the changes induced by sub-lytic complement activation results in endoplasmic reticulum (ER) stress [47,48], and one of the consequences of ER stress is the deficient intracellular clearance of misfolded proteins. This accumulation of misfolded proteins might result in autophagy and apoptosis, which can be prevented by upregulation of the ubiquitin–proteasome system (UPS) [49]. The UPS represents a coordinated system meant to degrade misfolded proteins, preventing the detrimental effects of protein accumulation. When ER-associated degradation is impaired, ubiquitinated proteins accumulate, reflecting the complementarity of the two protein-degrading systems [47]. In glomerular epithelial cells exposed to complement, the decrease in the ER protein degradation is paralleled by an increase in the overall availability of ubiquitinated proteins, suggesting the activation of the UPS as a reparative mechanism to prevent the accumulation of misfolded protein and podocyte injury [49]. In our study, we found upregulated UBE2K in FSGS, a member of the ubiquitin-conjugating enzyme family, with the main function of transporting ubiquitin from the ubiquitin-activating enzyme (E1) to the ubiquitin–ligase enzyme (E3). UBE2K plays an important role in determining whether a selected protein will be degraded [50].

The accumulation of ubiquitinated proteins has been observed in FSGS and in experimental MN [51,52,53]. When podocyte injury persists, UPS activation is associated with the degradation of FP and components of the podocyte cytoskeleton [41]. These modifications are not observed in transient podocyte injury. The accumulation of ubiquitinated proteins may indicate an overwhelmed UPS capacity. Observations of FSGS support the hypothesis that UPS activation is a reparative mechanism in persistent glomerular disease [42,50,54]. Interestingly, recent research has shown that the inhibition of a deubiquitinating enzyme in podocytes reduces proteinuria in MN, supporting this hypothesis [53].

We report here, for the first time to our knowledge, the upregulation of proteins involved in complement dysregulation and ubiquitination in human glomeruli from FSGS. Notably, both UBE2K and CFHR1 were upregulated in all but one patient.

The complement system links innate immunity to adaptative immune response. As shown in the string analysis (Figure 1 and Supplementary Files), 15 differentially expressed proteins in our study are associated with these aspects of immune response and have been reported to be interconnected (Figure 1). They also interact with proteins involved in pathways associated with structural or regulatory components of the cytoskeleton (TUBB, TUBB4B, and TUBA4A), FA (ITGB1 and COL4A1), cell adhesion (ITGB1, FBLN1, and PPIA) and ECM structures (ANXA2, COL4A18, and TINAGL1). The supplementary figures of the String analysis (Supplementary Figures S2–S4) showcase the differentially expressed proteins associated with the aforementioned pathways. However, although associated with the immune system, CFHR1 and UBE2K must be further studied in order to clarify interconnections to other molecules involved in the immune response.

One of the proteins that are upregulated in FSGS is cyclophilin A (PPIA), a member of the immunophilin family that exhibits peptidyl-prolyl isomerase activity. While it is primarily intracellular, it can also be expressed on the cell surface. In podocytes, it is localized at the level of focal adhesion; PPIA has been proven to exert pro-inflammatory actions and to participate in the immune response [55,56]. Cyclophilin A also serves as a binding site for calcineurin inhibitors and colocalizes with nephrin, podocalyxin, synaptopodin, and ß3-integrin. Through its binding to 14-3-3 protein, PPIA helps stabilize the cytoskeleton [57] which is essential for maintaining podocyte integrity and function.

It is noteworthy that 14-3.3 sigma, which is known to influence Rho GTP-ases and modify mainly primary foot process structure and FA [58], was found to increase in our FSGS patients. Yet a different variant of this molecule, namely 14-3-3 beta, primarily associated with tertiary FP, was increased in the MCD group. Similar differences in the expression of these proteins were described in experimental models and cell cultures [55]. Notably, one member of the Rho GTP-ase family, GNOA-1, was also found to be elevated in FSGS in our study.

Another structural protein with an important positive fold change in FSGS patients was TINAGL1, a matricellular protein that serves as a ligand for integrins, which, in turn, are important anchoring and signaling factors at the FA. TINAGL1 also binds to other structural matrix proteins, such as laminin, collagen, and fibronectin, being among the 10 most abundant proteins in the normal glomerular extracellular matrix. It is a glycoprotein present primarily in the mesangium, as well as in GBM, where it colocalizes with collagen IV alfa, laminin, and other matricellular proteins. Its role within the glomerulus remains to be defined, but it probably relates to the barrier function in glomerular disorder [58].

In what concerns proteins that are upregulated in MCD, the protein with the highest positive fold change is myosin light chain-3 (MYL3), part of myosin protein, known to be involved in cell motility and architecture by binding and exerting force on the actin filaments. Notably, MYL3 was previously found in immunohistochemistry studies in glomerular cells, and MYL3 transcripts were described to be associated with proteinuria [59]. Another highly expressed protein found is represented by neprilysin (MME), which is known to have kidney specificity according to the Human Protein Atlas database. Neprilysin stimulates angiotensin 1–7 induced reno-protective effects by interfering with angiotensin-1 and angiotensin-2 signaling. Podocytes are able to synthesize all the components of the renin–angiotensin system, angiotensin-2 is known to interfere with cytoskeleton regulation and angiotensin 1–7 may act as a mitigating factor in case of podocyte injury [60,61].

It would have been informative to compare proteomic profiles based on response to treatment, but given the limited number of patients in our study groups: two non-relapsing courses in the MCD group and two progressive courses in the FSGS group, statistical analysis of subgroup proteomic data could not be performed. Although some disparities in proteomic profiles might be suggested according to the course of disease, future tissue proteomic studies on larger cohorts are required in order to determine differences according to response to treatment; a prospective study in this regard is ongoing in our center.

Our study contributes significantly to the proteomic landscape of FSGS using human glomerular tissue, highlighting novel findings related to complement system dysregulation and protein ubiquitination mechanisms. These findings align with previous evidence implicating podocyte-driven complement activity and emphasize the need for further investigation of complement pathway involvement in FSGS. Future research should examine how complement activity interconnects with cytoskeletal and adhesion protein dynamics to drive glomerulosclerosis in order to fundament possible targeted therapies aimed at complement regulation. The consistent upregulation of ubiquitin-conjugating enzyme E2K in FSGS points to increased UPS activity, possibly reflecting a reparative response to persistent podocyte injury. Further studies are needed to validate the identified proteomic changes in larger, diverse patient cohorts using advanced immunohistochemical techniques.

## 5. Conclusions

Tissue proteomic studies most closely reflect the actual activated pathways, providing an accurate and nuanced understanding of the molecular processes that drive the disease. Our results not only deepen our understanding of podocyte-related diseases but also provide a basis for future investigations into targeted therapies. Given the current lack of comprehensive proteomic data in FSGS and MCD, we believe our study adds valuable information to the current incomplete map of proteomic studies in podocyte diseases.

## Figures and Tables

**Table 1:** Clinical characteristics of the patients included in the study.

Patient	MP1	MP2	MP3	MP4	MP5	MP6	GP7	GP8	GP9	GP10	GP11	GP13	MCD (*n* = 6)	FSGS (*n* = 6)	*p*-Value
Previous childhood NS-RC	Yes	No	No	Yes	No	No	No	No	No	No	No	No			
Previous immunosupression	Co/CNI	No	No	Co/CNI	No	No	No	No	No	No	No	No			
Sex (males), *n* (%)	F	M	M	M	F	M	F	M	M	F	F	M	4 (66.7)	3 (50)	1
Age (years) **	20	51	18	20	66	59	36	27	41	57	25	60	39 ± 22.07	41 ± 14.79	0.857
Serum creatinine (mg/dL) **	0.90	1.34	1.07	0.82	0.71	0.62	1.10	0.98	1.18	1.07	1.71	1.81	0.91 ± 0.26	1.31 ± 0.36	0.052
eGFR (mL/min/1.73) **	94	64	103	128	92	109	67	108	80	61	42	32	98.33 ± 21.23	65 ± 27.25	0.040
Serum albumin (g/dL) *	3.15	1.46	1.61	1.46	1.6	3.21	1.50	2.74	2.80	3.00	3.77	1.91	1.61 (1.46; 3.15)	2.77 (1.91; 3)	0.394
Total proteins (g/dL) **	5.82	3.44	3.89	3.38	4.2	5.82	5.00	5.03	5.45	5.82	6.41	5.08	4.43 ± 1.12	5.47 ± 0.56	0.070
Cholesterol (mg/dL) **	315	400	564	300	290	283	300	229	270	325	188	412	358.67 ± 109.23	287.33 ± 78.35	0.223
Hemoglobin (g/dL) **	15.1	15.6	18.1	14.9	12.0	11.6	15.1	13.9	14.5	16.2	12.9	11.2	14.55 ± 2.42	13.97 ± 1.75	0.643
Hematuria, *n* (%)	Yes	Yes	No	Yes	No	Yes	No	Yes	No	Yes	Yes	Yes	4 (80)	4 (66.7)	1
Proteinuria (g/24 h)	3.90	22.52	12.78	21.41	14.50	2.54	10.00	14.78	8.50	4.80	3.20	15.50	12.94 ± 8.44	9.46 ± 5.04	0.406
C3 (g/L) **	1.49	2.18	MD	1.78	1.82	1.33	1.12	1.21	1.52	1.52	1.60	1.64	1.72 ± 0.33	1.44 ± 0.22	0.117
C4 (g/L) **	0.42	0.51	MD	0.59	0.44	0.42	0.51	0.45	0.43	0.58	0.45	0.52	0.48 ± 0.07	0.49 ± 0.05	0.730
Immunosupresive Line 1	Co	Co	Co	Co	Co	Co	Co	Co	Co	Co	Co	Co			
Response to treatment	No	Yes	Yes	Yes	Yes	Yes	Yes	No	Yes	No	Yes	Yes			
Relapse	-	Yes	No	No	Yes	No	Yes		No	-	No	No			
Immunospresive Line 2	CNI	CFS	-	-	CNI	-	CNI	CNI	-	CNI	CNI	-			
Response to treatment	Yes	Yes	-	-	Yes	-	Yes	Yes	-	No	No	-			
Relapse	-	-	-	-	Yes	-	-	-	-	-	-	-			
Immunospresive Line 3	-	-	-	-	CFS	-	-	-	-	-	-	-			
Response to treatment	-	-	-	-	Yes	-	-	-	-	-	-	-			
Last follow up (months)	84	73	12	10	156	12	40	70	65	38	31	6			
Last known serum creatinine (mg/dL)	0.94	0.88	0.78	0.8	1.26	0.51	0.95	0.88	0	ESRD	ESRD	1.58			
Last known proteinuria (g/24 h)	1.6	0.19	0.12	0.24	0.31	0.4	0.58	0.54	0.3	ESRD	ESRD	0.42			

Legend: * median (25th—75th percentile); ** arithmetic mean ± standard deviation; qualitative variables (e.g., Sex) are presented as % of the group. Dichotomous variables were presented with absolute frequency (relative percentage frequency). eGFR—estimated glomerular filtration rate [24]; NS-RC—relapsing course nephrotic syndrome; Co—corticotherapy; CNI—calcineurin inhibitors; F—female; M—male; MD—missing data; CFS—cyclophosphamide; ESRD—end-stage renal disease.

## Data Availability

The data that support the findings of this study are available within the article and its Supplementary Materials. The LC-MS data used in this study are publicly available in the MassIVE repository under accession number MSV000097297, Pathogenesis of Focal Segmental Glomerulosclerosis and Minimal Change Disease (https://doi.org/10.25345/C5251FX92, accessed on 17 February 2025). Additional data are available from the corresponding author upon reasonable request, as they may require approval from the ethics committees of County Emergency Hospital Cluj-Napoca and “Iuliu Hațieganu” University of Medicine and Pharmacy.

## Supplementary Materials

The following supporting information can be downloaded at https://www.mdpi.com/article/10.3390/life15040527/s1. Figure S1: Differential expression analysis results in FSGS vs. MCD groups using a more stringent *p*-value cutoff; Figure S2: STRING PPI network and functional enrichment analysis results using the Gene Ontology Biological Process database of the differentially expressed proteins between FSGS and MCD; Figure S3: STRING PPI network and functional enrichment analysis results using the Gene Ontology Cellular component database of the differentially expressed proteins between FSGS and MCD; Figure S4: STRING PPI network and functional enrichment analysis results using the Gene Ontology Molecular function database of the differentially expressed proteins between FSGS and MCD; Table S1. Differentially expressed proteins (DEPs) in FSGS vs. MCD; Table S2. Characterization of differentially expressed proteins (DEPs) using the HPA database; Table S3. STRING functional enrichment analysis results of DEPs.

## Abbreviations

The following abbreviations are used in this manuscript:

FSGS	Focal segmental glomerulosclerosis
MCD	Minimal change disease
GBM	Glomerular basement membrane
UBE2K	Ubiquitin-conjugating enzyme 2K
CFHR1	Complement factor H-related protein-1
SD	Slid diaphragm
FP	Foot process
FA	Focal adhesion
FPE	Foot process effacement
MPGN	Membranoproliferative glomerulonephritis
FFPE	Formalin-fixed paraffin-embedded
TEM	Transmission electron microscopy
eGFR	Estimated glomerular filtration rate
RNase	Ribonuclease
DNase	Deoxyribonuclease
LCM	Laser capture microdissection
CNI	Calcineurin inhibitors
CFS	Cyclophosphamide
HPA	Human Protein Atlas
MAC	membrane attack complex
ECM	Extracellular matrix
FSGS-NOS	Focal segmental glomerulosclerosis not other specified
MAC	Membrane attack complex
UPS	Ubiquitin–proteasome system
ER	Endoplasmic reticulum
MN	Membranous nephropathy
CypA	Cyclophilin A
MYL3	Myosin light chain-3
MME	Neprilysin
DEPs	Differentially expressed proteins
PPI	Protein–protein interaction
FDR	False discovery rate
