# Supplementary material for: Pathogenesis of Focal Segmental Glomerulosclerosis and Minimal Change Disease: Insights from Glomerular Proteomics
Source: Life (Basel). 2025 Mar 23;15(4):527. doi: 10.3390/life15040527 (PMC12028441; doi:10.3390/life15040527)
Supplement: Supplementary file 1 [file life-15-00527-s001.zip › life-3511256-supplementary.pdf]

Supplementary Figures

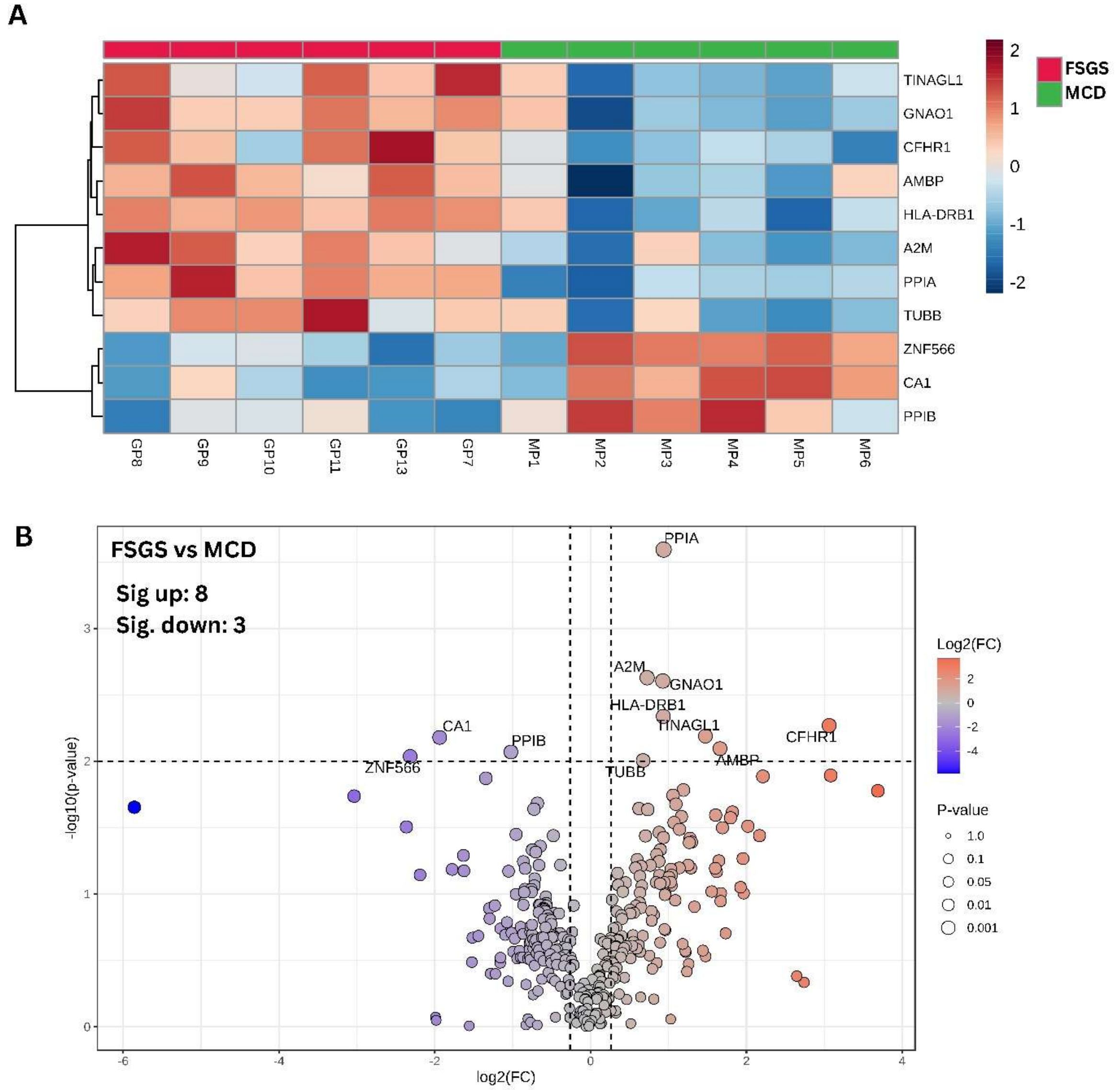

Supplementary Figure 1. Differential expression analysis results in FSGS vs MCD groups. A. Heatmap of differentially expressed proteins (unpaired t-test, unequal variance, $p \leq 0.01$; Euclidean distance measure) B. Volcano plot illustrating the differentially expressed proteins (t-test, independent unequal variance, $p \leq 0.01$ and $|FC| > 1.2$) in FSGS group compared to the MCD group. Red – DEPs with higher abundance in FSGS; Blue- DEPs with higher abundance in MCD; grey – proteins with no significant different abundance; gene name is provided for the top 10 DEPs

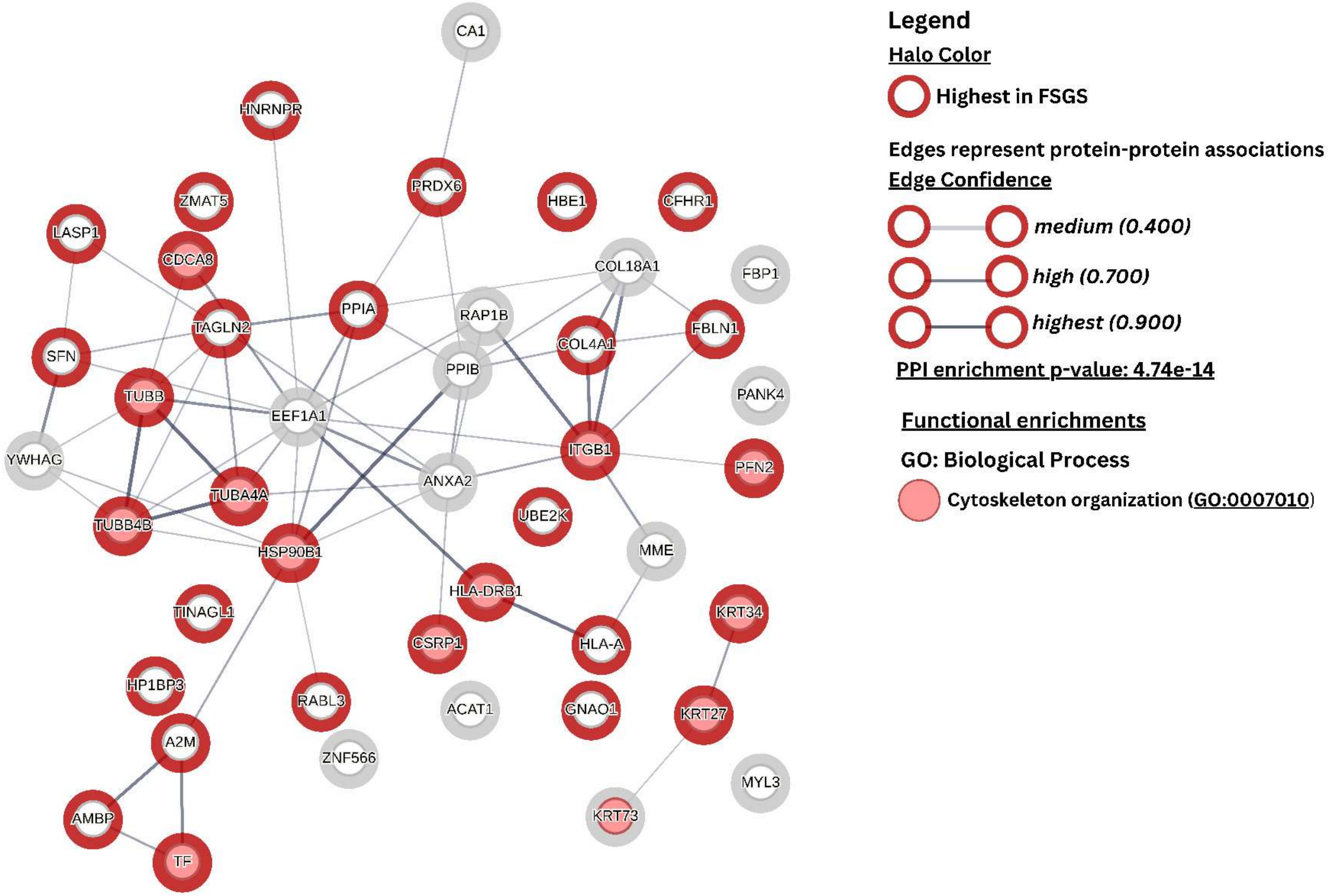

Supplementary Figure 2. STRING PPI network and functional enrichment analysis results using Gene Ontology Biological Process database of the proteins differentially expressed between FSGS and MCD.

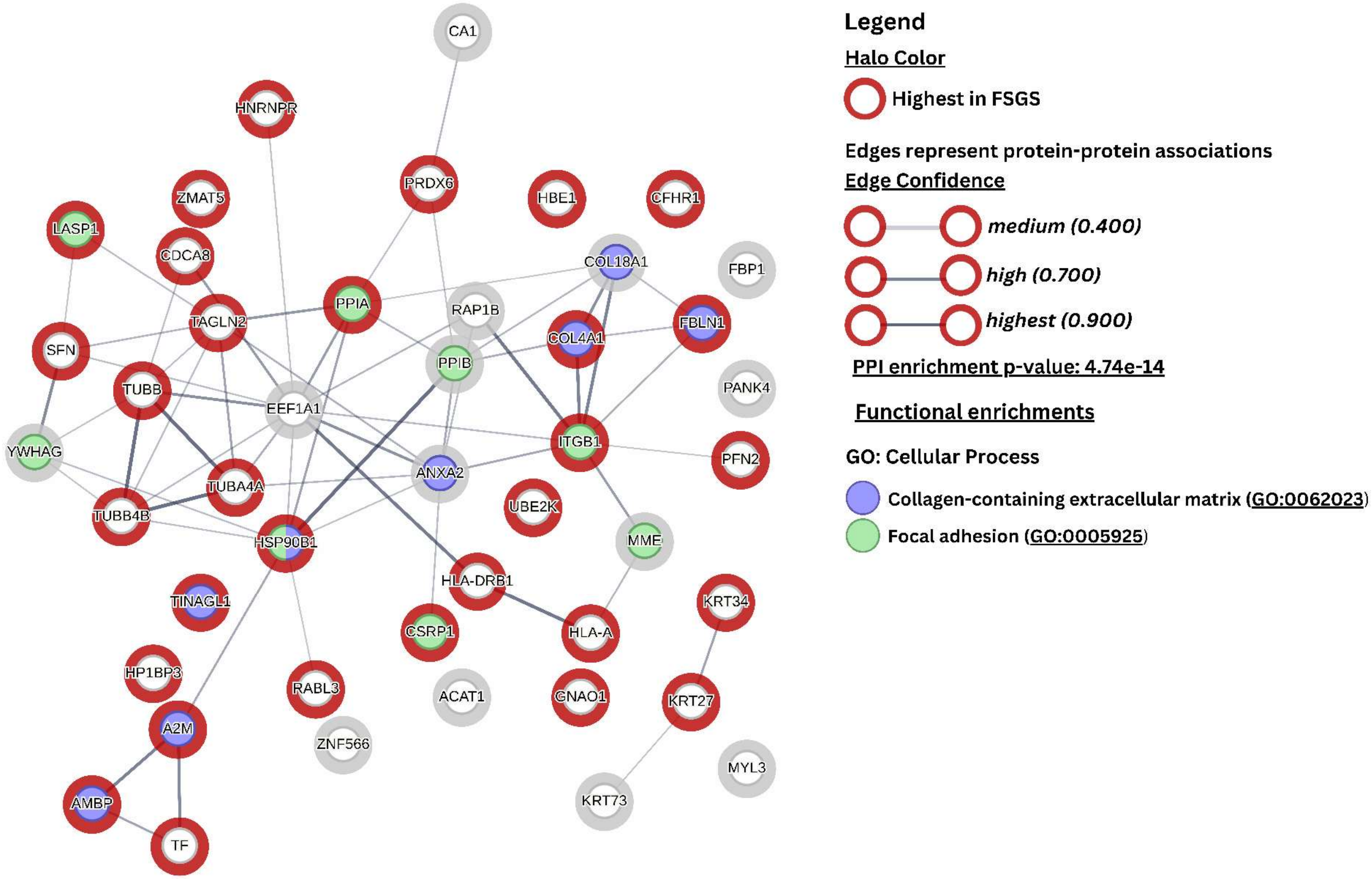

Supplementary Figure 3. STRING PPI network and functional enrichment analysis results using Gene Ontology Cellular component database of the proteins differentially expressed between FSGS and MCD.

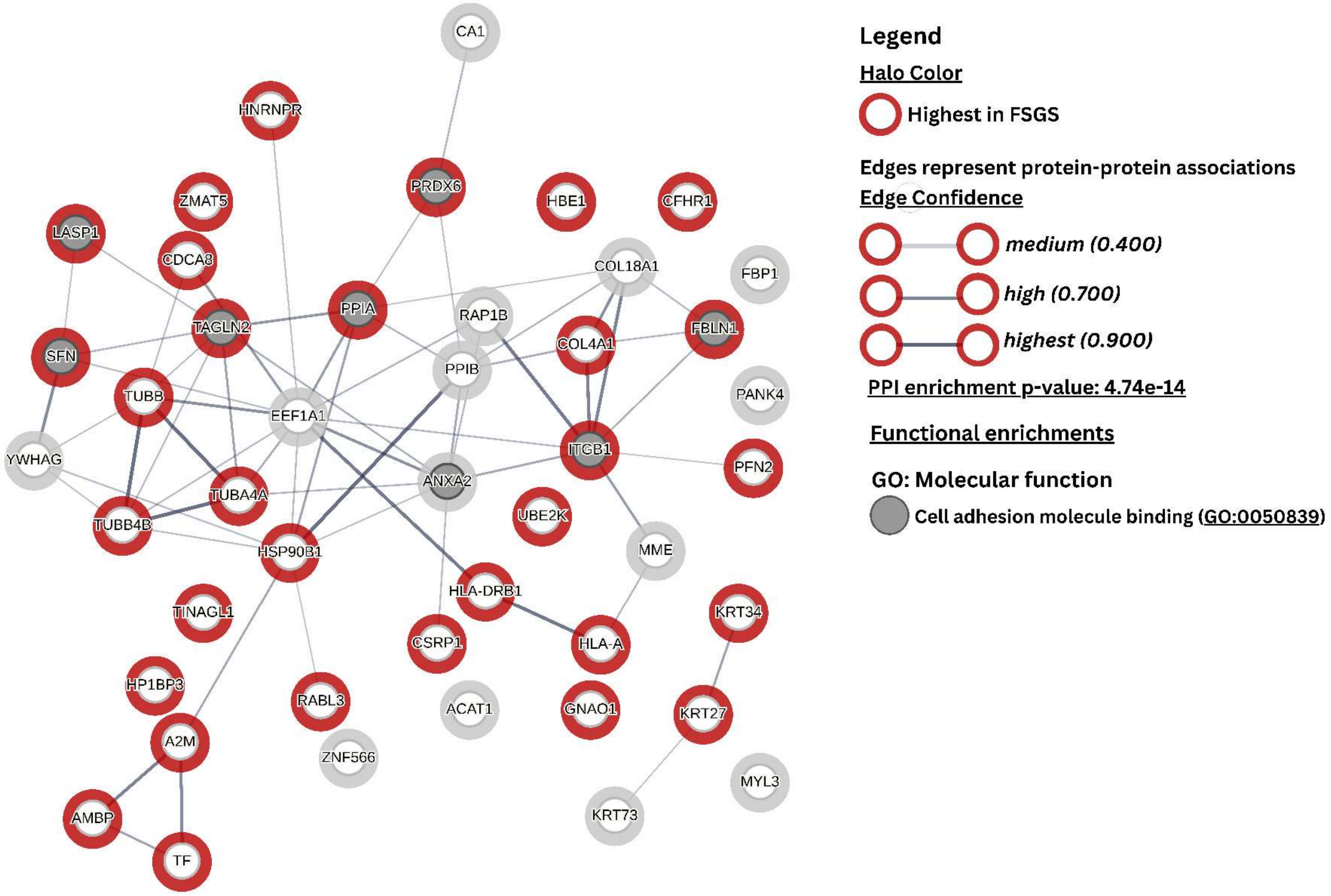

Supplementary Figure 4. STRING PPI network and functional enrichment analysis results using Gene Ontology Molecular function database of the proteins differentially expressed between FSGS and MCD.
